# A Consistent Lack of Consistency: Definitions, Evidentiary Expectations and Potential Use of Meaningful Change Data in Clinical Outcome Assessments Across Stakeholders. Results from a DIA Working Group Literature Review and Survey

**DOI:** 10.1007/s43441-024-00739-x

**Published:** 2025-01-10

**Authors:** M. Reaney, V. Shih, A. Wilson, B. Byrom, N. Medic, D. Oberdhan, C. Mamolo, M. Majumder

**Affiliations:** 1 IQVIA Patient-Centered Solutions, 3 Forbury Place, 23 Forbury Road, Reading, RG1 3JH, London, UK; 2 AstraZeneca, Gaithersburg, USA; 3 PAREXEL International, Waltham, NC USA; 4 eCOA Science, Signant Health, Nottingham, UK; 5 AstraZeneca, Cambridge, UK; 6 Otsuka Pharmaceutical Development & Commercialization, Inc., Rockville, USA; 7 Patient-Centered Outcomes Research, Genentech Inc., A Member of the Roche Group, South San Francisco, CA USA; 8 Bayer US LLC, Whippany, NJ USA

**Keywords:** Clinical outcome assessment (COA), Meaningful change, DIA working group, Literature review, Stakeholder survey, Within-patient change, Between-group difference, Patient engagement

## Abstract

**Background:**

Clinical outcome assessments (COAs) measure how patients feel or function and can be used to understand which patients experience benefits of treatment and which do not. Interpretation of COA data is influenced by how meaningful change is defined. We aimed to compare how different stakeholders define, assess, and use meaningful change for decisions that impact patients.

**Methods:**

A targeted literature review was undertaken in July 2021 using Medline, Embase, online grey literature search engines, and stakeholder organization websites. Additionally, a stakeholder survey on meaningful change was fielded between March and June 2023. Both quantitative and qualitative methods were used to analyze responses and identify key themes.

**Results:**

The literature review resulted in 86 references. These revealed different approaches to define, measure and validate meaningful change. There were 248 survey responses. Many respondents felt the terminology and methods for defining meaningful change are confusing. Respondents also emphasized the importance of distinguishing within-patient and between-group change, and defining meaningfulness from the patient perspective (most patients and caregivers do not share a similar definition of meaningfulness as their healthcare professionals).

**Conclusion:**

Four key recommendations for defining, establishing, and interpreting meaningful change estimates for COAs are: (1) Be clear on the type of “meaningful change” that is discussed or needed for a COA, (2) Ensure the “patient voice” is informing meaningful change estimates/definitions, (3) Acknowledge that a meaningful change estimate for a COA may differ between populations, diseases, and disease states, and (4) Disseminate data in a way that reduces ambiguity.

## Introduction

Clinical outcome assessments (COAs) represent an approach to support the measurement of clinical benefit, reflecting how a patient feels or functions. They are becoming an integral part of making approval decisions about an intervention at the regulatory and payer level [1] and are increasingly being used by healthcare professionals (HCPs) and patients when making care decisions [2]. For COAs to be maximally useful to regulators, payers, HCPs, and patients, it is important to be able to define what constitutes a meaningful change, both qualitatively and quantitatively [3]. Although researchers have been estimating meaningful change for over 20 years, it is still non-harmonized, made difficult by the plethora of definitions, variety of methods, and inconsistency in use of meaningful change data [4]. Additionally, commonly used relative measures of treatment effect estimates, such as a hazard ratio, do not readily translate to measurements on the patient level scale and adds to the challenge of defining and interpreting a meaningful change.

To address the issue of meaningful change in COAs, the Drug Information Association (DIA) Study Endpoints, Clinical Research, and Statistics & Data Science Communities launched four cross-community pre-competitive meaningful change working groups in 2021. One of the working groups (WGs) aimed to provide definitions and use of meaningful change terminology, another explored methods used to establish meaningful change thresholds with COAs, and a third aimed to define standards for evaluating meaningful change with digital health technologies (DHTs), specifically sensors and wearables. The final working group (WG2) aimed to consolidate definitions, evidentiary needs/expectations and potential use of meaningful change data among government regulators and policy makers, health technology assessment (HTA) agencies, health systems, payers, caregivers, patients, researchers, and biopharmaceutical companies.

WG2 conducted two research projects: (1) a targeted literature review focused on consolidating and presenting published methods related to deriving meaningful change thresholds as well as evidentiary needs/expectations and potential uses of meaningful change data in COA interpretation; and (2) a bespoke stakeholder survey focused on the same topics. Target stakeholders for the survey included: pharmaceutical/biotech professionals, HCPs, patients, health authority regulators, caregivers, and payer or HTA professionals. The findings of these research projects are described in this paper.

## Materials & Methods

### Literature Review

A targeted literature review was undertaken in July 2021 using Medline & Embase to explore meaningful change methods, approaches and uses as it pertains to COAs, to support activities across the three DIA cross-community meaningful change WGs.

Questions for the literature search were centered around definitions and use of meaningful change terminology (WG1), key stakeholder expectations and uses for meaningful change evidence (WG2), and methods used to establish meaningful change thresholds (WG3). The searches were restricted to research in humans published in the English language between 2016 and 2021. The search terms were adjusted to maximize completeness as well as feasibility for manual review. The final set of search terms and number of articles are shown in Table 1. The targeted literature review identified 3027 articles. The results discussed in this paper pertain to questions and findings on key stakeholder expectations and use of meaningful change evidence from WG2.

**Table 1:**
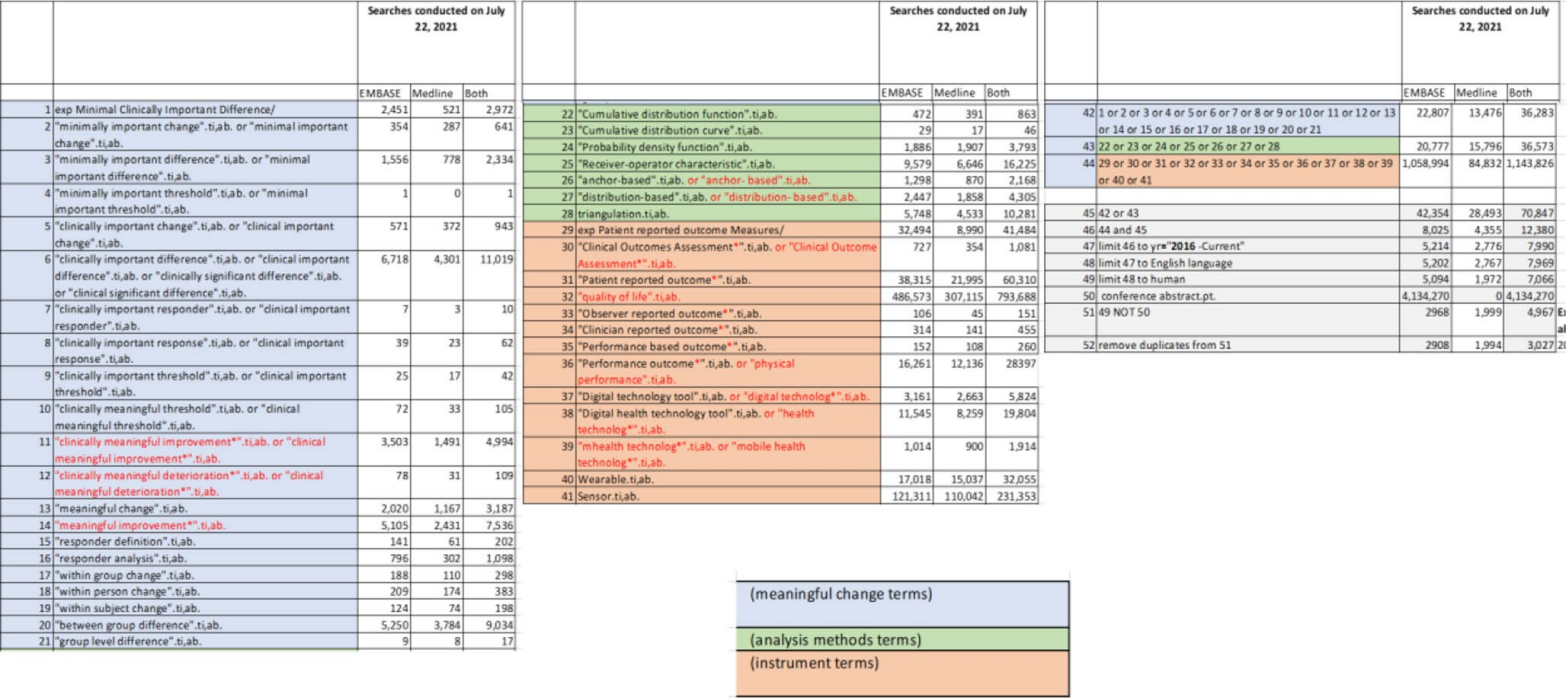
Search terms for the targeted literature review

Abstracts for these 3027 articles were screened for relevance and inclusion. The objective for WG2 was to identify articles outlining the aforementioned stakeholders’ evidentiary needs/expectations and potential uses of meaningful change data in COAs. Abstracts were tagged as relevant for WG2 objectives if they met at least one of the following criteria:

- Abstract has at least one author who is a regulator, policy maker, HTA, payer or health system representative, patient, or patient advocate.
- Abstract presents a paper that critiques the field/research around meaningful change and how it has been used.
- Abstract presents a paper that is making clear recommendations on meaningful change or expressing opinions/editorial comments.

Abstracts describing research where meaningful change was calculated and the study only reported on those findings were excluded.

A total of 475 articles (15.6%) were tagged as potentially relevant for WG2 based on initial abstract screening. These 475 articles were sourced and reviewed in full (where available) for relevance to the WG2 objectives. Following this, 49 articles (10.3%) were considered relevant and information from the full text articles was synthesized and summarized. A further 15 articles were added from reference lists or author-perceived relevance to the objectives (including some from before 2016).

**Fig. 1:**
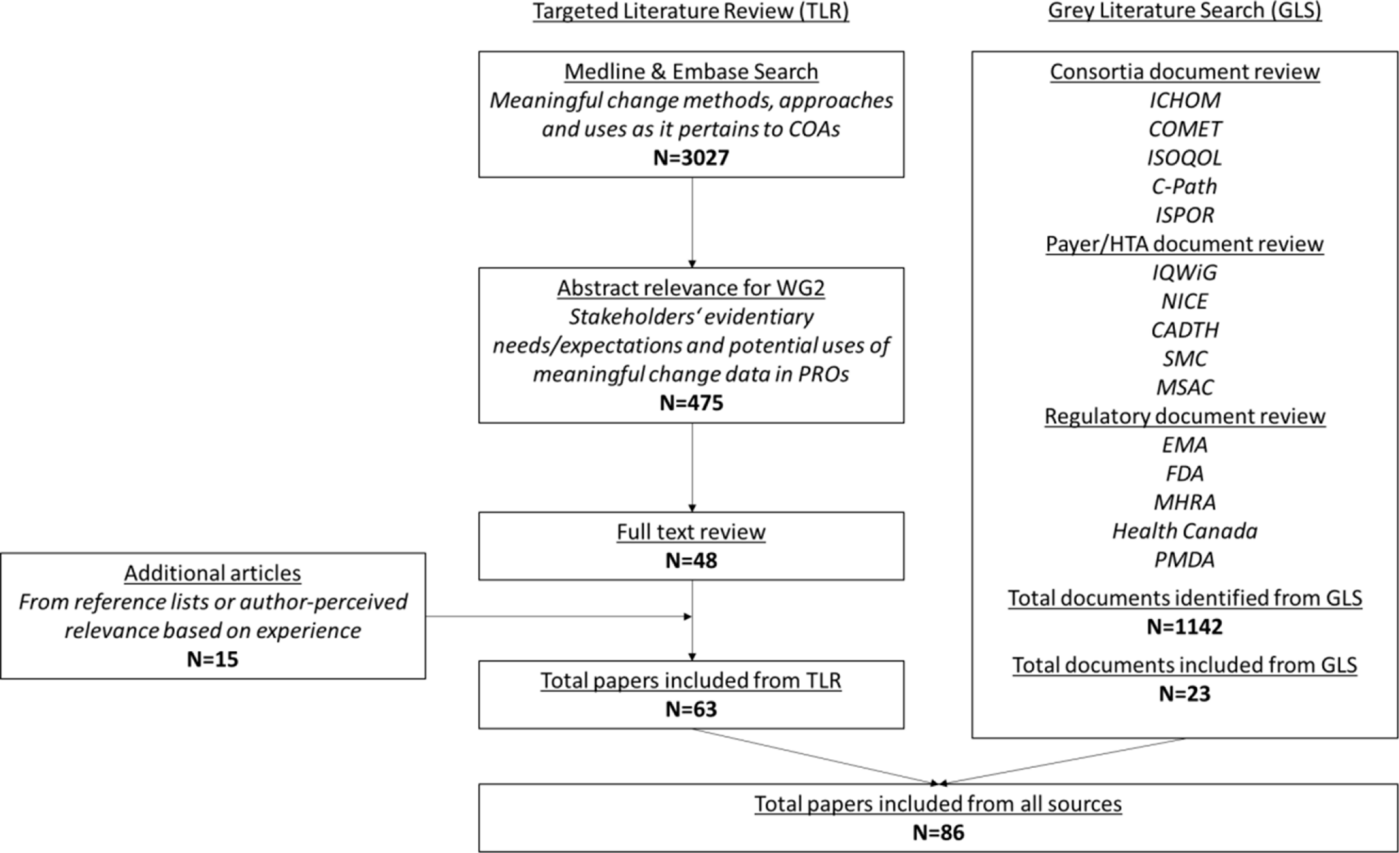
PRISMA flow diagram for the targeted literature review and grey literature search. CADTH = Canadian Agency for Drugs and Technologies in Health; COMET = Core Outcome Measures in Effectiveness Trials; C-Path = Critical Path Institute PRO Consortium; EMA = European Medicines Agency; FDA = Food and Drug Administration (USA); GLS = Grey Literature Search; ICHOM = International Consortium for Health Outcome Measures; ISOQOL = International Society of Quality of Life Research; ISPOR = The Professional Society for Health Economics and Outcomes Research; IQWiG = Institute for Quality and Efficiency in Healthcare; MHRA = Medicines and Healthcare products Regulatory Agency (UK); MSAC = Medical Services Advisory Committee (Australia); N = Number; NICE = National Institute for Clinical Excellence; PMDA = Pharmaceutical and Medical Devices Agency (Japan); SMC = Scottish Medicines Consortium; TLR = Targeted Literature Review; WG2 = DIA Meaningful Change Working Group 2

An additional grey literature search was completed using online search engines (e.g. https://greymatters.cadth.ca/) and organization websites to look at non-indication-specific guidance from consortia, payer/HTA groups, regulators and other sources known to be relevant to the question (e.g., International Consortium for Health Outcome Measures [ICHOM], International Society of Quality of Life Research [ISOQOL], the Professional Society for Health Economics and Outcomes Research [ISPOR]). This identified a further 23 relevant documents. A PRISMA diagram is shown in Fig. 1.

### Stakeholder Survey

A stakeholder survey on meaningful change was also developed to delve into current perspectives, definitions, and positions on evaluating COAs throughout the intervention development and decision-making processes. Each stakeholder answered a set of 29 to 34 questions about their background as well as their current thinking on meaningful change. Common questions were developed for all stakeholders to allow for comparable evaluations, but stakeholder-specific questions were also asked based on their expertise in the field. All responses were based on multiple choice options or free text.

DIA configured, advertised, and hosted the anonymous DIA Meaningful Change Working Group Survey using SurveyMonkey (SurveyMonkey Inc., San Mateo, CA) (https://www.surveymonkey.com/r/Preview/?sm=LH9rtauyBL04vo9DLza2DhWtrpzyUDfnqeckN1G115R45ZKztmzwLYURapZwEnK5) for open completion between 3 March 2023 to 16 June 2023. The survey was also disseminated via The Critical Path Institute, LinkedIn, and the DIA Breakfast Briefing. There were no restrictions to who could respond.

## Results

### Literature Review

The targeted literature review and the grey literature combined identified 86 documents relevant to the objectives of the review. From these documents there is some clear alignment but also some important differences.

**Fig. 2:**
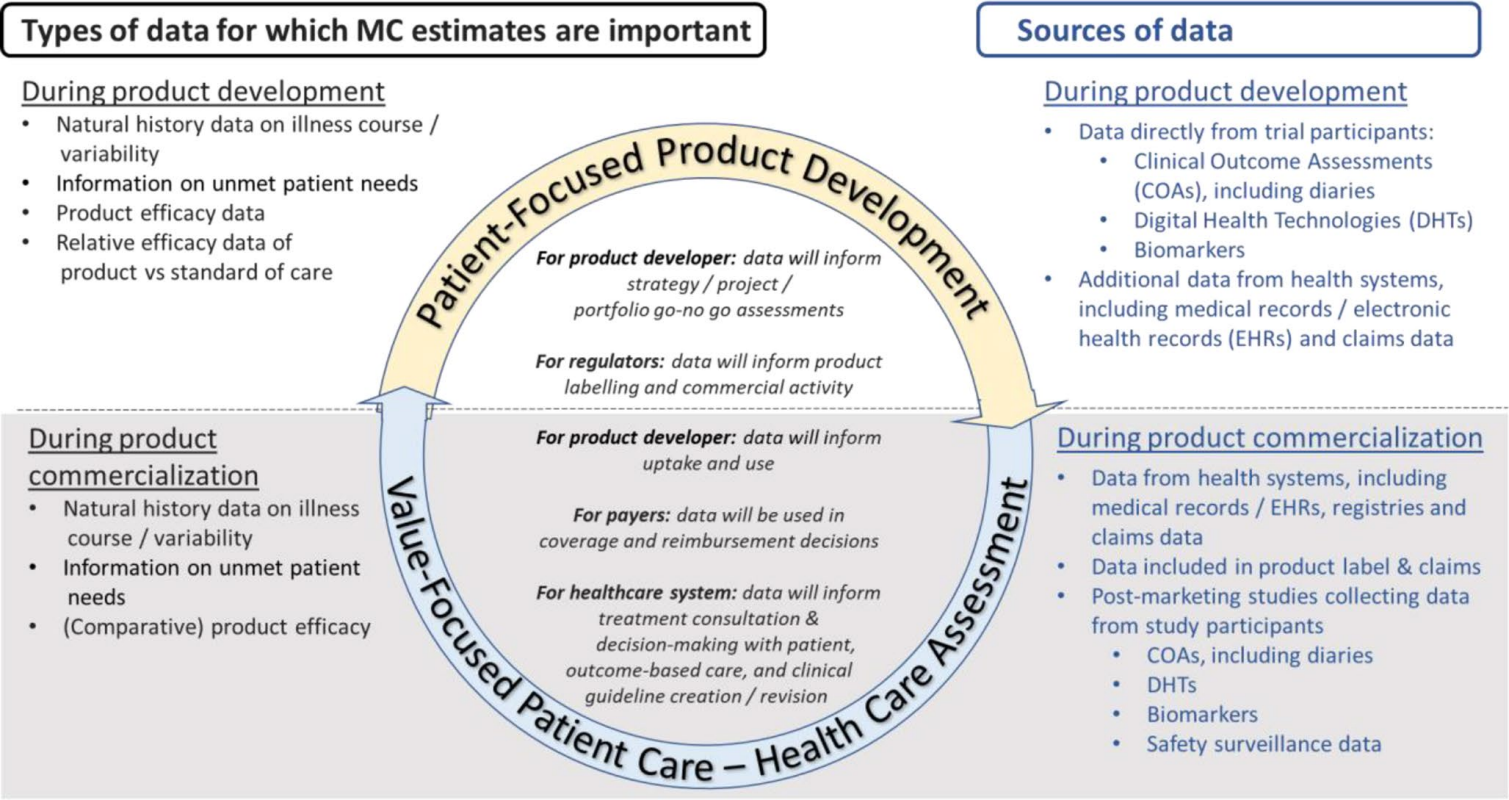
Use and sources of meaningful change data during intervention development and healthcare

There is a broad consensus across stakeholders that estimates of meaningful change are important for interpretation of COA data and help to expand understanding of treatment benefit beyond statistical significance alone. Having thresholds against which a change is considered meaningful can help design as well as aid interpretation and enhance the relevance of results in clinical trials [5, 6]. This is useful for intervention developers (sponsors) to determine natural history of disease, treatment effectiveness, and commercial viability, and is useful for regulatory review and interpretation of COA data, for example under the Patient-Focused Drug Development (PFDD) initiative at the US FDA and the Roadmap to 2025 initiative at the EMA [7, 8]. Further, consortia like the SISOQOL-IMI (Setting International Standards in Analyzing Patient-Reported Outcomes and Quality of Life Endpoints in Cancer Clinical Trials – Innovative Medicines Initiative) expert consensus group identify that both statistical significance and achieving a clinically meaningful difference is essential for drawing comparative conclusions [9]. In addition to traditional COAs measured through questionnaires, changes in COAs derived from digital health technologies (DHTs) and clinical biomarker data must be interpreted in the context of both clinical and patient relevance, and require definitions of meaningful change to support regulatory review. Having thresholds against which a change in COA, DHT or biomarker are considered meaningful can also facilitate payer review (including QALY interpretation and cost considerations [10, 11]), guideline development, and value-focused patient care, informing conversations, shared decision-making, and multidisciplinary management [12–16]. These uses are outlined in Fig. 2.

There is also agreement that thresholds for meaningful change are likely to vary according to culture and context, including demographic and disease considerations [3, 14, 17–19]. This means, for example, that published definitions of meaningful change in functioning COAs derived from a group of people who are mildly functionally limited may have limited value for studies that enroll people who are physically disabled [20]. However, it may be more nuanced than that – we may not be able to assume that the same definitions of meaningful change should be considered across research and clinical practice settings, or even within a research population [3].

The documents reviewed offered discussion about three different types of meaningful change: meaningful within-person change over time, meaningful within-group changes over time, and meaningful between-group differences at a specific timepoint. Some articles present data using general terms like “minimally important differences” and “clinically relevant change” without specifying which type of meaningful change they are primarily focused on. This inconsistent use of terminology is problematic in both appraising the literature and conducting research on meaningful change [21]. However, guidance from regulators [3] and payers/HTAs [22], and articles with regulators and payer representatives as authors (e.g [23]), offer some specificity. They are primarily focused on meaningful within-person change and/or meaningful between-group differences.

Meaningful within-person change can be defined as “the amount of change an individual person would have to report to indicate that a relevant treatment benefit or worsening has been experienced” [24]. This change should be one that “has clinical or practical importance [and] has an impact on an individual’s self-perceived health status or quality of life” [25]. Meaningful within-person change is often referred to in the literature as a ‘responder definition’ or a ‘clinically important response’ (CIR) [26]. Meaningful between-group differences refer to the differences in (change) scores between treatment groups that can be considered relevant. “It can be used as a threshold to define whether one group represents a relevant benefit over another at a specific timepoint or in terms of change over time” [24]. A meaningful between-group difference is often referred to in the literature as a ‘minimally important difference’ (MID) or a ‘clinically important difference (CID)’ [26]. Advisory groups and consensus statements in areas like asthma [27] and interstitial lung disease (ILD) [28] also consider interpreting data in these ways, and recent evidence further suggests that patients and healthcare professionals have an interest in being presented with data showing the proportion of people who benefit from treatment (i.e. a meaningful within-person change) [29–33].

There have been calls to standardize approaches to present meaningful change data from clinical trials. These have focused on meaningful within-person changes, and propose reporting the proportion of people who improved, worsened or stayed about the same following a clinical intervention [34, 35] to aid interpretation of between-group differences. But the literature remains inconsistent in clear terminology, type of meaningful change calculated, and presentation of data. The SISAQOL-IMI is a public-private consortium formed to establish a harmonized set of consensus recommendations on, among other topics, the definitions, use, and interpretation of meaningful change in oncology. Their recommendations are expected over the next few years and may be applicable to areas outside of oncology as well.

Further, there is quite some variability in perceptions of whether meaningful changes should be defined using clinical parameters, or patient perspectives [36, 37]. Some stakeholders, such as clinical consensus groups, think about meaningful change in clinical terms; that is, they tie a meaningful change in a COA score to a clinical parameter, to ascertain what level of change in a COA score is required for a clinical benefit to be assumed/realized. Other stakeholders, such as FDA, consider COAs in patient terms; that is, they tie a meaningful change in a COA score to a perceived improvement by the patient in their health or functioning. Indeed, the importance of the patient-focused perspective to the FDA is evident in their development of four guidance documents focused on enhancing incorporation of the patient’s voice into drug development and regulatory decision-making [7]. A recent review found that out of 41 studies using anchor-based methods, 36 studies applied patient-reported anchors and only 5 studies applied clinician-reported ones [38]. Where research has been conducted using both clinical and patient-reported anchors, data do not always harmonize into a single value, or even a narrow range of values [38, 39]. There are also notable differences of opinion in whether the focus should be on “minimally” important changes, “important changes” or “acceptable states” [40, 41]. The traditional focus has been on minimal change [18, 42], although this is now changing [3, 43]. More recently it has been proposed to distinguish between people who had a large improvement/worsening, and a small improvement/worsening [44].

Finally, there is no real consensus on the most appropriate way of calculating meaningful changes [21]. Two commonly used techniques are the distribution-based and anchor-based methods [42, 45]. Distribution-based methods use a statistical approach, such as defining meaningful change in a COA as being larger than one-half standard deviation in size, while anchor-based methods determine meaningful change in reference to surveys of patients’ own impressions of benefit or satisfaction or clinical markers of treatment benefit. Aside from the Institute for Quality and Efficiency in Health Care (IQWiG) in Germany that defines a change threshold of 15% as acceptable (‘a small but noticeable change’) if no data are available to determine a meaningful change threshold for a specific endpoint, regulatory and payer bodies typically do not accept meaningful change threshold determination based on distribution-based methods alone. The FDA, for example, have stated a clear preference for anchor-based approaches [3] as distribution-based approaches do not directly take into account the patient voice, and tend to give lower values than the anchor-based approaches [3, 46]. Others have used the receiver operating curve (ROC) methodology as a way of establishing a meaningful change estimate (e.g [18]).

In summary, the literature review highlighted several challenges for intervention developers as well as other decision makers as to how to build evidence for meaningful change thresholds that will be useful for approval & beyond Marketing Authorization. These primarily derive from a lack of harmonization across stakeholders.

## Stakeholder Survey

A total of 248 stakeholders responded to the meaningful change survey: 155 (62.5%) were pharmaceutical/biotech professionals, 52 (21.0%) were HCPs, 20 (8.1%) were patients, 9 (3.6%) were health authority regulators, 7 (2.8%) were caregivers, 5 (2%) were payer or HTA professionals (Table 2). The respondents were all adults above 18 years of age, with an average age of 49 years. The majority were female, Caucasian, from the United States/North America or the European Union and had post-graduate degrees (Table 2). Most of the HCPs were from a university or academic center, or a public hospital; more than half of the industry professionals worked in a pharmaceutical company; and caregivers tended after children, adults, and the elderly. Disease severity was reported as mainly moderate or severe by the caregiver or patient themselves, with an average of 25 years with the disease.

**Table 2:** Demographics of Stakeholder Survey respondents

Parameter	*n* (%)
Stakeholder type	
Caregivers	7 (2.8%)
Health authority regulators	9 (3.6%)
HCPs	52 (21.0%)
Patients	20 (8.1%)
Payer or HTA professionals	5 (2.0%)
Pharmaceutical/biotech professionals	155 (62.5%)
Age	
18–30 years old	4 (3.1%)
31–40 years old	27 (20.8%)
41–50 years old	39 (30.0%)
51–60 years old	31 (23.8%)
61–70 years old	19 (14.6%)
71–80 years old	4 (3.1%)
71–90 years old	0 (0.0%)
>90 years old	0 (0.0%)
Prefer not to answer	6 (4.6%)
Sex	
Female	88 (68.0%)
Male	37 (28.0%)
Non-binary	0 (0.0%)
Prefer not to answer	5 (3.8%)
Race	
Asian or Asian American	18 (14.0%)
American Indian or Alaska Native	0 (0.0%)
Black or African American	2 (1.6%)
Native Hawaiian or other Pacific Islander	0 (0.0%)
White or Caucasian	104 (80.6%)
Other	5 (3.9%)
Geographic region	
Africa	0 (0.0%)
Asia / South Asia / Southeast Asia	5 (3.9%)
Australia / New Zealand	1 (0.8%)
European Union	36 (27.9%)
Latin America / Caribbean	3 (2.3%)
United States / North America	84 (65.1%)
Education level	
Did not complete mandatory education	0 (0.0%)
Completed school but did not engage in higher education (college/university)	0 (0.0%)
Completed an undergraduate degree program at college/university	15 (11.5%)
Completed post-graduation education (after undergraduate degree)	114 (88.5%)
Other	0 (0.0%)
Prefer not to answer	1 (0.8%)

Percentages are based on the total number of stakeholders who responded to the survey question

**Fig. 3:**
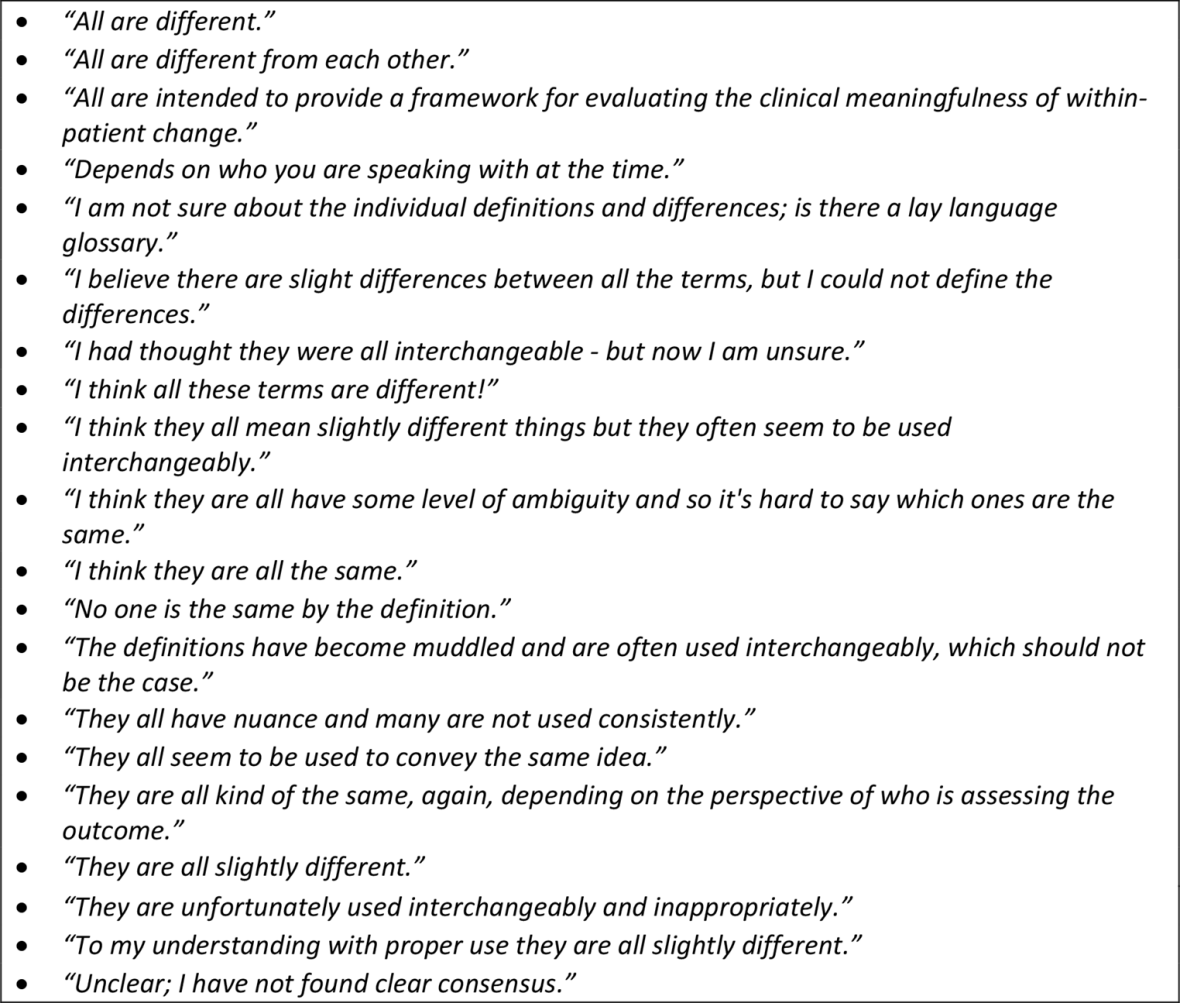
Quotes on Overall Similarities and/or Differences in Meaningful Change Terminology: MCT, MID, MCID, CMC, Responder Definition

Industry, HCP, and regulatory stakeholders were asked if they thought ‘meaningful change’ is well-defined, and 83% of the respondents said no, 10% said yes, and 7% had no opinion. They were also asked which meaningful change terms they have heard or used in the past, indicating whether they had heard the terms meaningful change threshold (MCT), minimal important difference (MID), meaningful clinically important difference (MCID), clinically meaningful change (CMC), and responder definition. Survey results show that 31% reported hearing or using MCID, followed by CMC (22%), MCT (17%), responder definition (14%), then MID (10%). Many thought MCT, MID, MCID, CMC, and Responder Definition were equivalent, although others considered them as different (Fig. 3). These stakeholders highlighted the importance of understanding within-patient versus between group differences, with some respondents indicating that ‘responder definition’ and MCT are commonly used to refer to within-patient change while MCID is often used to describe between group differences, but the terminology used to make this distinction varied.

Respondents were asked to indicate their preferences for the terms and explain why. The term MID was not preferred by many, as it was considered *“out of date”* due to the focus on minimal change; *“anything ‘minimal’ is not useful, as it does not equal what is meaningful”*. Meaningfulness is inherent in the CMC and MCT titles and therefore these were preferred. However, CMC was reported as an *“umbrella term”* that *“doesn’t necessarily define a threshold”* and *“takes it back to the hands of clinical assessments”* which does not account for patient perspectives. The word ‘clinical’ in MCID also elicited strong opinions on the implied lack of patient perspectives:

*I don’t like because it takes it back into the hands of clinical assessments, the purpose of this should be to evaluate from the patient perspective.*

*I don’t like using the term ‘clinically’ as it could be confused with something that is relevant for clinicians, while it is NOT only but rather should be significant for patients themselves.*

*… remove the paternalistic clinical terminology [‘clinically’]. And who cares if [it is] meaningful to [a] clinician – [it] needs to be meaningful to [the] patient.*

**Fig. 4:**
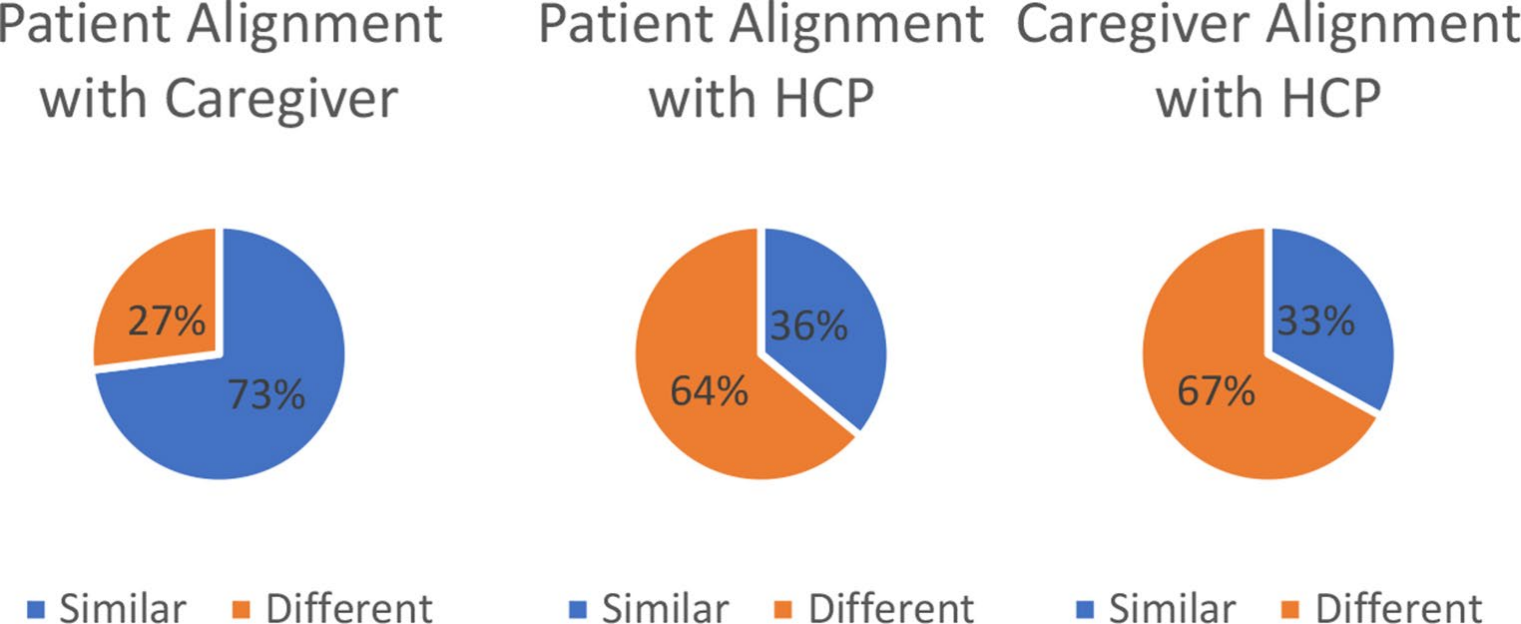
Level of Alignment in Patient Perception of Definition of Meaningfulness Compared to Caregivers and Healthcare Providers a: Patients perceptions (*n* = 11) of whether their definition of meaningful change is similar or different to their caregivers^1^. b: Patients perceptions (*n* = 11) of whether their definition of meaningful change is similar or different to their healthcare team c: Caregivers perceptions (*n* = 3) of whether their definition of meaningful change is similar or different to the healthcare team^3. 1^ Responses from patients (*n* = 11) to the question “Do you think that how you define meaningful (in terms of improvement or worsening of your disease/condition) is the same as how your loved ones/the person you care for defines meaningful?” (yes/no) ^2^ Responses from patients (*n* = 11) to the question “Do you think that how you define meaningful (in terms of improvement or worsening of your disease/condition) is the same as how your healthcare team provider(s) defines meaningful?” (yes/no) ^3^ Responses from caregivers (*n* = 3) to the question “Do you think that how you define meaningful (in terms of improvement or worsening of the disease/condition of the person you care for) is the same as how their healthcare team provider(s) define meaningful?” (yes/no)

These comments align with the discordance on what is considered ‘meaningful’ for patients and their caregivers versus clinicians. When patients and caregivers were asked how their definition of ‘meaningful improvement or worsening’ compared to that of their HCPs, 73% of patients think their caregivers define meaningfulness the same as they do but only 36% of them share similar definitions with their HCPs. Similarly, only 33% of caregivers think HCPs share the same definition of meaningfulness as their loved ones (Fig. 4). There was broad agreement that meaningful change should be defined from the patients’ perspective and the terms ‘responder definition’ and MCT were considered to be more patient focused. They were also described as *“overarching”*, *“up-to-date and usually more clearly defined”*, *“used more with PRO experts”* and *“easier to understand by cross-functional colleagues”*.

Respondents were also asked how meaningful change estimates should be derived. 78% of industry and regulators both prefer estimates to be generated using both quantitative and qualitative methods, as opposed to 20% based on quantitative methods only and 2% based on qualitative means only. Half of the HCPs and regulators considered that meaningful change estimates should consider duration of disease, although how to do so would depend on the disease. When asked what meaningful improvements would be for them or the patient(s) they care for, HCPs, patients, and caregivers all mentioned improvements in symptoms and mental health as critical components. HCPs and patients also considered clinical assessments (including medication use), quality of life, prevention of disease progression or survival, and overall perception as important; and HCPs and caregivers also mentioned needs for expanded access to existing health diagnostics, care, and access.

## Discussion

Over the past several years, there has been an increasing interest in and use of meaningful change as an outcome of interest in clinical trials and other aspects of healthcare and patient-centered research [47]. With this has come debate on the types of clinically meaningful change and their uses as well as confusion regarding terminology. DIA WG2 undertook a literature review and stakeholder survey in order to ascertain the current state of use of different clinically meaningful change approaches among various stakeholders (pharmaceutical/biotech professionals, HCPs, patients, health authority regulators, caregivers, payers, or HTA professionals).

The literature review showed that while there are three main types of meaningful change (within-person change, between-group change, and within-group change); the type is often not identified in the literature and is described with overlapping terminology. The context in which the meaningful change threshold was established and with which methods is important to help readers understand whether the intent was to define within-person, within-group or between-group change, and to determine if the threshold can be used to compare research results. The confusion resulting from use of varying terminology in the literature is reflected in the survey results where the most notable point of agreement between the stakeholder survey findings and literature review results was the confusion regarding terminology, in particular the variety of terms and how they are frequently used interchangeably (even when not appropriate). Most stakeholders agreed the definition of meaningful change was not well-defined and recognized the importance of distinguishing within-patient versus between-group change. While the two are not mutually exclusive, they aren’t interchangeable [48]. Some decision makers utilize meaningful change information to help interpret presented p-values for treatment group differences often presented with group means, while others prefer within-patient-based meaningful change information as part of responder analyses.

Additionally, the survey uncovered strong respondent opinions on the term “clinical” or “clinically” in commonly used terminology (e.g., MCID), with a suggestion that meaningfulness should be defined from the perspective of the patient rather than through clinical means. Indeed, the word “clinical” in the definitions was interpreted to reflect a disregard for the patient perspective while focusing instead on clinical assessment. A discordance was noted between the goal of considering what is meaningful to patients while using clinician instead of patient-focused terminology. This was not identified in the literature review, but was further supported by most patients, as well as caregivers in the survey, who reported they do not share a similar definition of meaningfulness as their HCPs. For this reason, the terms ‘responder definition’ and MCT were preferred over the others.

The lack of specificity in communications, regarding settings and use cases when it comes to terminology causes ongoing confusion and problems with messaging and interpretation. The issue is further complicated by a lack of consensus on methods to establish meaningful change thresholds and ranges, which is compounded by a range of anchors used for anchor-based analyses. Based on the survey, responder definitions or MCT estimates for within-person and between-group change should be based on both quantitative (anchor-based, distribution-based) and qualitative evidence, which is consistent with draft FDA guidance [3]. The survey further highlighted that various other disease-related and personal factors (such as symptoms, mental health, ease of access, quality of life) should be considered in the definition of meaningful improvement or worsening.

Both the literature review and survey have several limitations. The literature review was conducted while important regulatory guidance documents in the field are under development and stakeholders are determining how to determine meaningfulness of results for their use case. While the survey was open for completion to any of the five stakeholders, findings may not be representative of all perspectives and ideas given the short duration for completion (approximately 3 months) and the ability for respondents to skip any question, leading to missing data. The survey included a broad variety of stakeholders (pharmaceutical/biotech professionals, HCPs, patients, health authority regulators, caregivers, payer or HTA professionals), although a limitation of the survey is that most of the responses (63%) were from participants in the pharma/biotech industry, with few responses from other groups (HCPS, caregivers, patients, HTAs/payers and regulatory groups). It is possible there would be notable differences between these different stakeholders, however a larger sample would be needed to draw any firm conclusions. The majority of responses were from participants in North America with fewer responses from people in other areas of the world. While similar efforts to study clinically meaningful change are ongoing by other groups like SISAQOL-IMI, those tend to be solely focused on a single therapeutic area (e.g., oncology). However, one key strength of this research is its broad scope. The literature review and survey were not limited to any particular disease areas and were open to participation from a variety of stakeholders. The literature review considered both published literature and grey sources from various stakeholders, with 23 documents found from regulatory agencies and HTA/payers. Although there was limited survey participation from these groups, the findings from the grey literature strengthened the evidence from these perspectives. The findings from our literature review and survey are therefore generalizable across disease areas, and of relevance to anyone with an interest in this field.

An important limitation of this study is its limited focus on causal attribution in the context of meaningful change. While the distinction between treatment effects and other contributing factors, such as natural disease progression or random variability, is acknowledged, this paper does not aim to fully address methodologies for isolating causal effects. Future research should explore frameworks and analytical approaches that incorporate causal inference to better contextualize meaningful change estimates, particularly within clinical trials [49, 50]. These efforts will help bridge the gap between stakeholder-driven definitions of meaningfulness and robust causal effect estimation, ensuring that meaningful change metrics are both interpretable and actionable across diverse settings.

## Conclusion

The broad scope and findings from the literature review and the stakeholder survey highlight 4 key points for researchers to consider when defining, establishing, and/or interpreting meaningful change estimates for COAs in the future.

1. Be clear on the type of ‘meaningful change’ that is discussed or needed for a COA, including whether causal effects are accounted for. Determine and specify whether the meaningful change estimate pertains to within-group, between-group, or within-patient change, and where possible distinguish changes attributable to treatment effects from those likely driven by other external factors.
2. Ensure the “patient voice” is informing meaningful change estimates/definitions. Commonly used anchors such as “clinician global impression” (CGI) or “physician global assessment” (PhGA) can only provide information about meaningfulness from the clinician perspective. Use of a patient-reported anchor (such as the Patient Global Impression scale [PGI] or Patient Global Assessment [PGA] scale; ideally using a short recall) allows for incorporation of the patient perspective, which is preferred if the concept can be easily understood by patients. The degree of change for an anchor measure considered meaningful will depend on the studied population and the response options of the anchor. Qualitative evidence from patients can support meaningful change thresholds and interpretation, including the appropriateness and meaningfulness of clinician- and patient-reported anchor measures.
3. Acknowledge that the meaningfulness of a specific level of change in a COA score may vary depending on disease state, population characteristics, and baseline values. As an example, a within-patient meaningful change threshold for the Montgomery-Åsberg Depression Rating Scale (MADRS) may be as high as 10 points in a treatment-resistant depressed population with MADRS mean baseline score of 37 [51] but as low as 8 points in a major depressive disorder (MDD) population with a MADRS mean baseline score of 28 [52].
4. Disseminate data in a way that reduces ambiguity. Until the field aligns on terminology, authors and stakeholders should clearly state which terms they are using and their respective definitions. As a variety of terms have been used in the past, the term “COA score interpretation threshold” may be the most appropriate for future use.

Our findings underscore the continued complexity of defining and interpreting meaningful change in COAs, particularly when causal effects are not explicitly accounted for. Future research should prioritize methodologies to enhance the validity and interpretability of meaningful change estimates, addressing both patient and stakeholder needs.

## Data Availability

No datasets were generated or analysed during the current study.
